# Utilizing the KSC Fixation Tube to Conduct Human-Tended Plant Biology Experiments on a Suborbital Spaceflight

**DOI:** 10.3390/life12111871

**Published:** 2022-11-13

**Authors:** Natasha J. Haveman, Mingqi Zhou, Jordan Callaham, Hunter F. Strickland, Donald Houze, Susan Manning-Roach, Gerard Newsham, Anna-Lisa Paul, Robert J. Ferl

**Affiliations:** 1 Department of Horticultural Sciences, University of Florida, 2550 Hull Road, Fifield Hall, Gainesville, FL 32611, USA; 2 Plant Molecular and Cellular Biology Program, University of Florida, 2550 Hull Road, Fifield Hall, Gainesville, FL 32611, USA; 3 Aegis Aerospace Inc., Kennedy Space Center, Merritt Island, FL 32889, USA; 4 Consolidated Safety Services, Inc., Merritt Island, FL 32953, USA; 5 Interdisciplinary Center for Biotechnology Research, University of Florida, 2033 Mowry Road, Gainesville, FL 32610, USA; 6 Office of Research, University of Florida, 1523 Union Rd, Grinter Hall, Gainesville, FL 32611, USA

**Keywords:** suborbital space, KSC fixation tubes, RNA-seq, arabidopsis, transcriptomics

## Abstract

Suborbital spaceflights now enable human-tended research investigating short-term gravitational effects in biological systems, eliminating the need for complex automation. Here, we discuss a method utilizing KSC Fixation Tubes (KFTs) to both carry biology to suborbital space as well as fix that biology at certain stages of flight. Plants on support media were inserted into the sample side of KFTs preloaded with RNAlater in the fixation chamber. The KFTs were activated at various stages of a simulated flight to fix the plants. RNA-seq analysis conducted on tissue samples housed in KFTs, showed that plants behaved consistently in KFTs when compared to petri-plates. Over the time course, roots adjusted to hypoxia and leaves adjusted to changes in photosynthesis. These responses were due in part to the environment imposed by the encased triple containment of the KFTs, which is a requirement for flight in human spacecraft. While plants exhibited expected reproducible transcriptomic alteration over time in the KFTs, responses to clinorotation during the simulated flight suggest that transcriptomic responses to suborbital spaceflight can be examined using this approach.

## 1. Introduction

The commercial suborbital spaceflight industry enables customers to reach the cusp of space and experience microgravity for several minutes before returning to Earth. The two main companies at the forefront of suborbital space efforts are Blue Origin (BO) and Virgin Galactic (VG). The two companies use different vehicles for their suborbital launches. Blue Origin flies New Shepard, a vertical rocket with a Crew Capsule. Virgin Galactic flies SpaceShipTwo, a rocket plane that is carried to high altitude before being dropped for launch. As such, the two providers present different modes of operations, different customer experiences and different flight profiles. Common features of both suborbital providers are a high g launch, minutes of microgravity conditions, followed by a high g re-entry. Although, the providers have different flight and gravity profiles, they both enable approximately three minutes of unmitigated microgravity [1,2]. It is those minutes of microgravity that are of high interest to science.

From a biological researcher’s perspective, three minutes of high-quality microgravity opens the possibility to conduct a myriad of experiments—including but not limited to testing hardware capability, validating hypothesis, and understanding biological responses of not only microgravity, but the entirety of the spaceflight profile. Even though providers currently offer researchers an opportunity to send payloads on suborbital flight, there are severe limitations in that the untended experiment payloads are required to be contained in a locker, under fully autonomously controlled without the ability of the scientist to monitor and adjust for unforeseen situations. Thus, human suborbital spaceflight provides a promising complementary opportunity for researchers to conduct human-tended microgravity-related experiments using human capabilities that can better recapitulate a laboratory bench experiment than can an automated locker payload. Sampling biological responses at various phases of flight for subsequent analysis would be a natural initial experiment to understand biology responses to suborbital space.

Plants being sessile in nature have evolved mechanisms to rapidly respond to their environment. Early transcriptomic responses have been investigated in response to various stresses such as; oxidative stress, heat stress, pathogen attack, and water stress [3,4,5,6,7,8,9]. Suborbital flights allow for the investigation of how plants quickly respond to transitions in varying gravity forces. To conduct such an experiment, there are several parameters that need to be considered. The rapid transitions into and out of microgravity occur within minutes, thus there is limited time for sample preparations and fixation. Plants should be poised and ready in a medium that confers as little confounding stresses as possible and upon exposure to the changes in gravity, and then be easily and rapidly preserved for proper biochemical analysis. Samples and fixatives should be triple contained and compact in size so that the hardware can be easily stowed during the flight. In addition, the acts of human tended fixation of the samples should be able to be completed rapidly.

This paper describes a ground-based experiment setup that was developed to meet the needs for conducting a human tended plant biology experiments in a suborbital flight. This method highlights the expanded use of KSC Fixation Tubes as both houses for biology and for the subsequent fixation of that biology. The paper also discusses best practices for both experiment setup and conducting a RNAseq analysis to reliably report on the transcriptome of plant samples at various stages of a suborbital flight profile.

## 2. Materials & Methods

### 2.1. Plant Growth Conditions

Wet-sterilized Columbia-0 (Col-0) seeds were planted aseptically on the surface of 10 cm^2^ solid media plates comprised of the coupon inlaid with 1% Phytagel/0.5× MS media. Plants were grown under a broad-spectrum LED light bank (100 µmoles/m^2^) for approximately 24-h the first three days. On day 3, plates were moved to dark storage (to simulate travel to launch locations) where they spent 8 h, before being transferred to a growth unit (Danby #DFG17A1B, Montreal, QC, Canada) which is used at remote locations for plant growth. The Danby provided 24 h of approximately 80 µmoles/m^2^ of light for another 4 days.

### 2.2. Plant Dissection and RNA Extraction

From each condition, two individual plants were pooled for RNA extraction and a total of three biological replicates were obtained from two coupons in each KFT. Leaves, hypocotyls, and roots from each set were dissected under an Olympus dissecting microscope (Olympus, Tokyo, Japan) for downstream applications, whereas the intervening hypocotyls were set aside. RNA extraction was performed using Qiagen RNeasy Plant mini kit (Qiagen, Hilden, Germany catalog #74904) according to the manufacturer’s guidelines. RNA concentration was determined on Qubit 2.0 Fluorometer (Thermo Fisher Scientific, Waltham, MA, USA) and RNA quality was assessed using the Agilent 2100 Bioanalyzer (Agilent Technologies, Inc., Santa Clara, CA, USA).

### 2.3. RNA-Sequencing Library Preparation

First, 100 ng of total RNA was used for mRNA isolated using the NEBNext Poly(A) mRNA Magnetic Isolation Module (New England Biolabs, Ipswich, MA, USA, catalog #E7490). Then, followed by RNA library construction with the NEBNext Ultra II Directional RNA Library Prep Kit (New England Biolabs, catalog #E7760) with 2/3 of poly A enriched RNA and 1/10 reaction volume using SPT Labtech mosquito LV liquid handling instrument. Briefly, RNA was fragmented in NEBNext First Strand Synthesis Buffer via incubation at 94 °C for the desired time. This step was followed by first-strand cDNA synthesis using reverse transcriptase and random hexamer primer. Synthesis of ds-cDNA was performed using the 2nd strand master mix provided in the kit, followed by end-repair and adaptor ligation. At this point, Illumina adaptors were ligated to the sample. Finally, each library (uniquely barcoded) was enriched by 15 cycles of amplification, and purified with Agencourt AMPure beads (Beckman Coulter, catalog #A63881). Barcoded libraries were sized on the Bioanalyzer and quantified with the Qubit 2.0 Fluorometer. Finally, these individual libraries were pooled in equimolar concentration for sequencing aiming for 60 million reads per sample with total of 1 lane of NovaSeq S4 2 × 150 cycles run. RNASeq libraries were constructed at the UF ICBR Gene Expression Core (https://biotech.ufl.edu/gene-expression-genotyping/, accessed on 11 November 2022. RRID:SCR_019145). The Illumina NovaSeq 6000 was used to sequence the libraries for 2 × 150 cycles. Sequencing was performed at the ICBR NextGen Sequencing (https://biotech.ufl.edu/next-gen-dna/, assessed on 11 November 2022. RRID:SCR_019152).

### 2.4. Illumina NovaSeq6000 Sequencing

Normalized libraries were submitted to the “Free Adapter Blocking Reagent” protocol (FAB, Cat# 20024145) in order to minimize the presence of adaptor-dimers and index hopping rates. The library pool was diluted to 0.8 nM and sequenced on one S4 flow cell lane (2 × 150 cycles) of the Illumina NovaSeq6000. The instrument’s computer utilized the NovaSeq Control Software v1.6. Cluster and SBS consumables were v1.5. The final loading concentration of the library was 120 pM with 1% PhiX spike-in control. One lane generated 2.5–3 billion paired-end reads (~950 Gb) with an average Q30% ≥ 92.5% and Cluster PF = 85.4%. FastQ files were generated using the BCL2fastQ function in the Illumina BaseSpace portal.

### 2.5. Bioinformatics Pipeline and Gene Ontology Analysis

Reads acquired from the Illumina NovaSeq 6000 platform were cleaned up with the cutadapt program [10] to trim off sequencing adaptors and low-quality bases with a quality phred-like score < 20. Reads <40 bases were excluded from RNA-seq analysis. The genome of Arabidopsis thaliana (version TAIR10.51) from TAIR (The Arabidopsis Information Resource) was used as the reference sequences for RNA-seq analysis. The cleaned reads of each sample were mapped to the reference sequences using the read mapper of the STAR package (Spliced Transcripts Alignment to a Reference, v2.7.9a) [11]. The mapping results were processed with the HTSeq (High-Throughput Sequence Analysis in Python, v0.11.2) [12] samtools, and scripts developed in house at ICBR of UF to remove potential PCR duplicates, choose and count uniquely mapped reads for gene expression analysis. The counted reads of each gene were analyzed by a DESeq2-based R pipeline. Significant up- and down-regulated genes were selected using the threshold of −1.0 < Log2FoldChange < 1.0 and *p*-value ≤ 0.05. Gene Ontology (GO) analysis was performed using g:GOSt (https://biit.cs.ut.ee/gprofiler/gost, accessed on 11 November 2022.) with default settings to obtain terms of GO biological process.

## 3. Results and Discussion

### 3.1. Optimizing Plant Growth Conditions within the KFT

**Figure 1:**
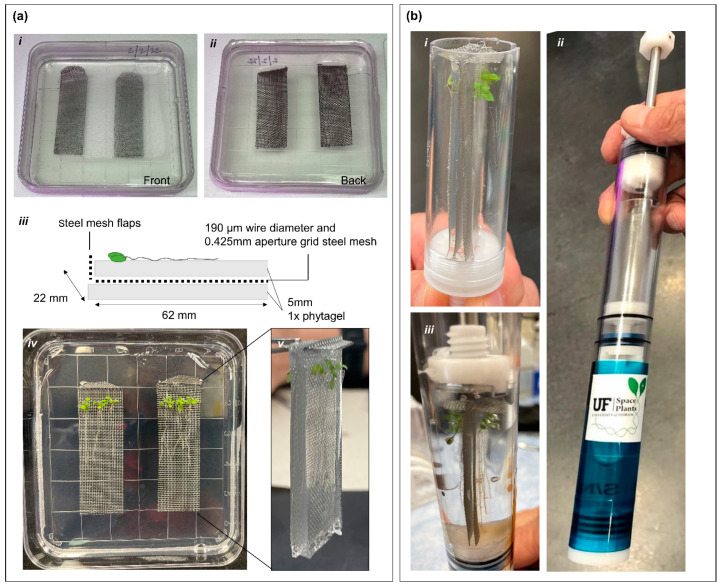
Preparation of Arabidopsis plant coupons within the KFTs for human-tended suborbital spaceflight. (**a**) Illustration of the various steps in making the plant coupons. Wire mesh coupons were encased in the solidified phytagel media (*i*) front (*ii*) back. (*iii*) schematic of the coupon layout. (*iv*) Image of the plants growing on coupons inlaid in phytagel matrix. (*v*) Cut out of the coupons placed together back-to-back for insertion into KFTs. (**b**) Placement of coupons into KFTs. (*i*) Shows coupons inserted into the sample area of the KFTs. (*ii*) Image of pre-actuated KFT. (*iii*) Image of actuated KFT with samples fixed in RNAlater.

The original purpose of the KFT was to allow astronauts in space to apply fixative or other chemical compounds that are often toxic to biological samples without the use of a microgravity glovebox while maintaining three levels of containment [13]. For example, KFTs have been used in the past to fix plant samples from a number of spaceflight plant biological experiments [14,15,16,17,18]. The dimensions of the sample compartment of the KFT (Figure 1a) were 22.1 mm (diameter) × 62.8 mm (depth).

The general application has been that the astronaut would remove a biological sample from its growth container and transfer the biology to the KFT sample tube. Once the sample was in the KFT, the KFT was sealed, and the fixative was brought into contact with the sample by activating the KFT mechanism [15]. However, for use in the short timeframes of suborbital flights, the KFTs need to be sealed before flight. This requirement necessitates that the biology be installed into the KFTs as part of a preflight procedure, and the KFT therefore becomes a biology incubator before it is activated to fix the sample. This allows the KFT to serve its purpose of containment safety for the entirety of the suborbital flight.

Many previous plant molecular biology spaceflight-related experiments utilized Petri dishes containing a matrix nutrient gel to grow plants on the ISS in a variety of plant growth hardware systems (i.e., BRIC, Biological Research in Canister; ABRS, Advanced Biological Research System; EMCS, European Modular Cultivation System; Veggie). Typically, these plates are taped closed with a porous surgical Micropore^®^ 3M™ tape, that allows for gas exchange, allowing plants to grow in this configuration for up to fourteen days.

To ensure that plants are kept healthy, intact, and experience minimal stress responses when deployed into the KFTs, plants were grown for 7 days on wire mesh coupons suspended into growth media within the 100 mm square Petri plate. The details to construct the deployable coupon are as follows. First, 0.425 mm stainless steel wire mesh (Yikai, UPC: 615435104645) was cut into 22 mm by 62 mm sections, with one short end rounded and bent for holding. The mesh coupons were then autoclaved. The square Petri plates were poured with 10 mL of media, allowed to slightly cool, and then the wire mesh coupons were laid on the solidified media. Finally, 15 mL of media was added to the dish to cover the tops of the coupons. Sterilized seeds were planted approximately 5 mm below the bend in the coupons and were grown vertically to allow roots to grow down the coupons (Figure 1a). When ready for placement into KFTs, the media and coupons with plants on the surface were cut out and carefully placed into the KFT (Supplementary Video Data S1). As the internal diameter of the KFT’s sample chamber is the same size as the width of the coupons, the edge of the entire length and base of the coupon is supported by the KFT resulting in an exact fit. Each KFT can hold two coupons, back-to-back, with a total of six 7-day old Arabidopsis plants on each. The bottom chamber of the KFT is filled with ~25 mL of RNAlater and upon actuation of the plunger, RNAlater flows into the sample chamber and the fixation of the plants is immediately initiated [14,15].

**Figure 2:**
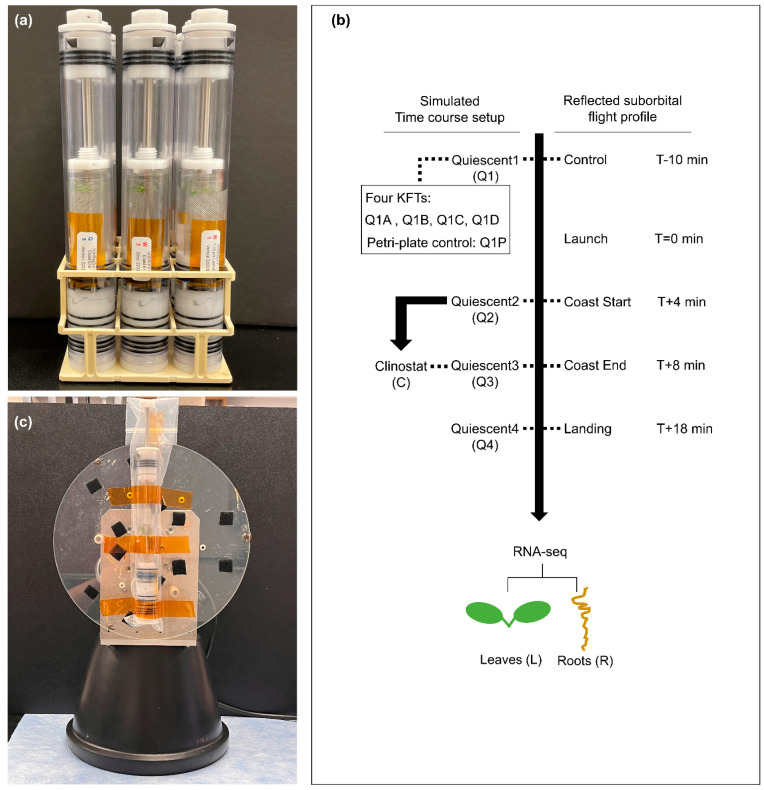
Setup of KFTs for simulated suborbital spaceflight profile. (**a**) Image of KFTs loaded with coupons and actuated after the respective timepoints. (**b**) Four time points of quiescent (Q1–Q4) and one set of treatment on clinostat (C) from Q2 to Q3, seedlings were harvested. Another set of four KFTs were used for comparison with a Petri dish control at Q1. The tissues were fixed in RNAlater overnight and then dissected to roots and leaves, which were subjected to RNA-seq analysis. (**c**) Image of KFT mounted on a 2-D Clinostat.

A simulation of a simple suborbital flight profile was used in this ground-based study to investigate the responses of plants grown in the KFTs over a time period roughly similar to the flight profiles for both Blue Origin and Virgin Galactic. To initiate pre-flight sample preparation, plants on coupons were inserted into the experiment KFTs, the KFTs were sealed by inserting the actuators, and all KFTs were placed in the vertical orientation (Supplementary Video Data S2). This simulates the preparation of biology into the KFTs to be ready for transport and installation into the spacecraft. KFTs were that left in the vertical orientation and not disturbed through the time course were referred to as Quiescent (Q), (Figure 2). After the KFT’s were set up and sealed, they were left to rest for 3 h and 50 min, to model a reasonably long time prior to launch. During actual launch operations, this would the time that the samples would be required to undergo check-out and hand off to vehicle loading procedures for flight operations.

Samples at Q1 (T-10 min, 3 h 50 min after sealing in the KFTs) were actuated and fixed 10 min before the mock launch timepoint (T+0). Additionally, at the T-10 min timepoint, an additional set of four KFTs (Q1A, Q1B, Q1C and Q1D) were actuated to assess the variability and reproducibility of the plant transcriptome simply from being housed for that amount of time in the KFTs. To understand how this methodology differs from the standard Petri dish configuration typically used in spaceflight experiments, the plant transcriptome obtained from each of the four Q1 KFTs (Q1A-D) were compared to the transcriptome from a Petri dish control at the same T-10 min timepoint.

Through the remainder of the time course, one KFT was used for each timepoint. Samples Q2, Q3, and Q4 were actuated 4 min, 8 min, and 18 min after T+0 mock launch, which mimicked the flight profile times of boost end / coast start (Q2: T+4 min), coast end (Q3: T+8 min), and landing (Q4: T+18 min), respectively. In addition, a set of samples were placed on a clinostat starting at the Q2 timepoint and fixed at the Q3 timepoint. Here, the KFT was mounted onto a 2-dimensional clinostat parallel to the plane of rotation (Figure 2c) and was rotated at 1 rpm for 4 min. Based on the radius (150 mm) of the clinostat, a rotation at 1 rpm would result in a simulated altered microgravity profile of 1.67 × 10^4^ g.

KFTs were actuated, filling the sample compartment with RNAlater. Plants were allowed to imbibe in RNAlater at room temperature for 24 h. After fixation, seedlings were harvested at each timepoint and each KFT, with the plants on the two coupons within each KFT divided into three biological replicates. For each sample, roots and leaves were subjected to RNA-seq analysis.

### 3.2. KFT Provides a Suitable Microenvironment for Short-Term Plant Growth

**Figure 3:**
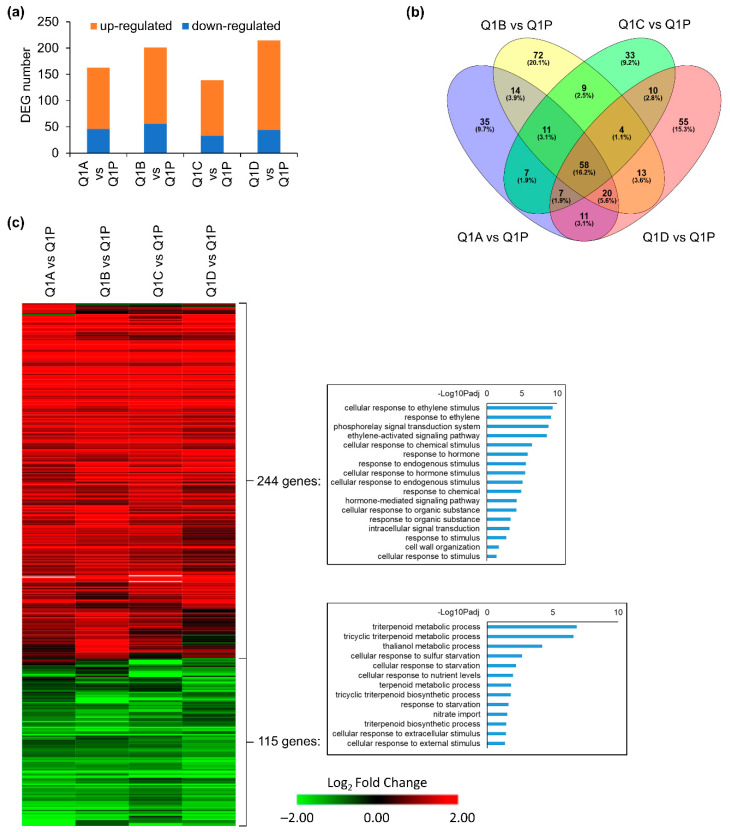
DEGs of KFT (Q1A-D) vs. plate control (Q1P) in roots at Q1. (**a**) Number of DEGs in four comparisons. (**b**) Overlap between DEGs detected in each comparison. (**c**) Heatmap showing expression patterns of 359 DEGs detected in at least one comparison. GO terms of biological processes for upregulated and downregulated genes are shown. Hierarchical clustering of heatmap was done using one minus cosine similarity. Genes with no expression detected in RNA-seq are indicated in gray.

Root tissues from four replicate KFTs (Q1A-D) and one Petri dish (Q1P) were analyzed to investigate how plants responded to living within the KFTs for three hours and fifty minutes, in order to assay the effects of preflight sealing into the KFTs. The four KFTs showed a total of 163, 201, 139, and 178 Differentially Expressed Genes (DEGs), when compared to Q1P, the samples taken directly from the Petri plates (Figure 3a). The composition of DEGs in the Q1A-D KFT replicates compared to Q1P showed a substantial number of similarities, with 45.7% of DEGs shared by at least two comparisons of KFT versus Q1P Petri plate (Figure 3b). In addition, a pairwise comparison between replicates showed an average of only 23 DEGs (Table 1), suggesting that growth conditions with each of the KFTs had little variation. This observation of reproducibility across four individual KFT replicates was also highlighted in the trends observed in the gene expression heatmap (Figure 3c, Table S1). Across all four KFT replicates, DEGs that were significantly altered in at least one comparison had the same regulatory trend across all the KFTs. This is indicated by the consistent Log fold change coloration in any given gene row of the heatmap. Gene Ontology (GO) analysis of the list of 244 DEGs upregulated in root tissue grown in KFTs compared to the plate showed an enrichment for ethylene response.

To be compliant with the triple containment criteria for operation of liquids in a suborbital flight, the KFTs must be sealed, and it is expected that plants placed within the KFTs will not have gas exchange. Therefore, an upregulation of ethylene is probable when compared to plants grown on the Q1P Petri plate. In the 115 downregulated DEGs, GO enrichment showed that cellular responses associated with nutrient starvation were repressed. These physiological responses, particularly ethylene accumulation, are commonly observed for plants grown in hardware used during spaceflight [19]. These data suggest that when compared to the typical Petri dish growth for flight experiments, the KFTs can serve as a containment vessel that allows plants to stay healthy over a several-hour duration as researchers prepare for a suborbital flight. There were gene expression differences in the plants living in the KFTs compared to those living on Petri plates, but that is expected and confirmed by these data.

### 3.3. Plant Responses to KFTs over Time in a Simulated Suborbital Flight Profile

**Figure 4:**
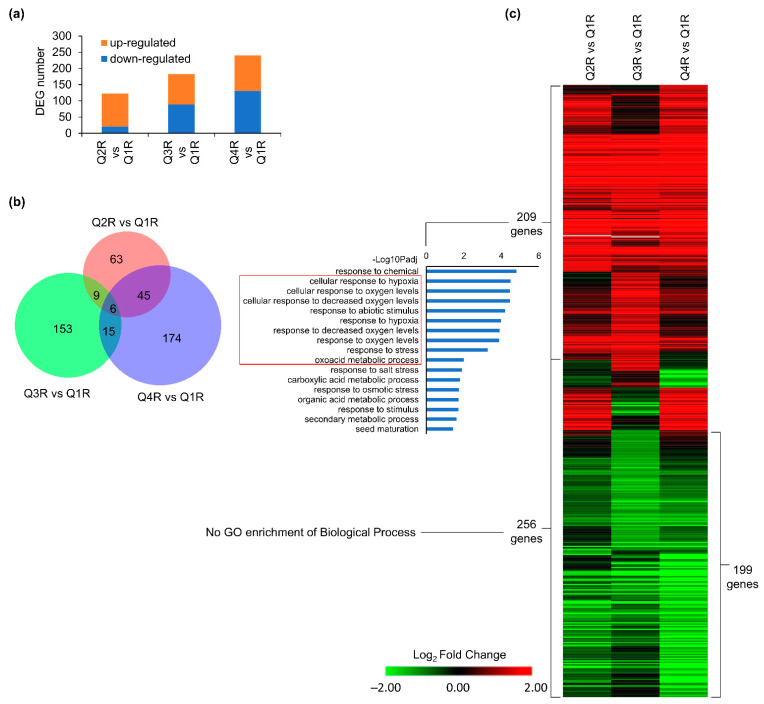
DEGs detected in at least one comparison of Q2, Q3 or Q4 vs. Q1 in roots. (**a**) Number of DEGs in three comparisons. (**b**) Overlap between DEGs detected in each comparison. (**c**) Heatmap showing expression patterns of 465 DEGs detected in at least one comparison. GO terms of biological processes for 209 induced genes are shown. Hierarchical clustering of heatmap was done using one minus cosine similarity. Genes with no expression detected in RNA-seq are indicated in gray. The red box highlights GO enrichments that are associated with hypoxia responses.

The intent of the time-course experiment that modeled a suborbital flight profile was to investigate plant transcriptomic profiles in the KFTs over the time it takes to complete a suborbital flight, and to access how to best establish ground controls. KFT’s were loaded and sealed at T-4 h prior to the T+0 timepoint. Root tissues (R) harvested from the KFTs actuated at coast start (Q2R), coast end (Q3R), and landing (Q4R) were compared to the control Q1R (T-10 min) In roots, there was an increase in the number of DEGs over time with 123, 183, and 240 DEGs reported for Q2R vs. Q1R, Q3R vs. Q1R, and Q4R vs. Q1R, respectively (Figure 4a). Interestingly, more than 50% of all the reported DEGs were unique to each time point (Figure 4b). This observation suggests that over time, different genes in the root tissues were engaged or repressed to acclimate growing in the KFT environment. However, trends in the gene expression pattern (Figure 4c, Table S2), demonstrate genes in all three time points behaved similarly. GO analysis of the upregulated genes showed that genes associated with hypoxia were enriched across all three time points. Analysis of the 256 DEGs including the 199 downregulated genes across all time points showed no significant GO enrichment associated with biological processes (Figure 4c). By comparing plant responses in KFTs for different durations to the KFT vs. plate study, it validates that the absence of gas exchange is the dominant response root tissues adapt to.

**Figure 5:**
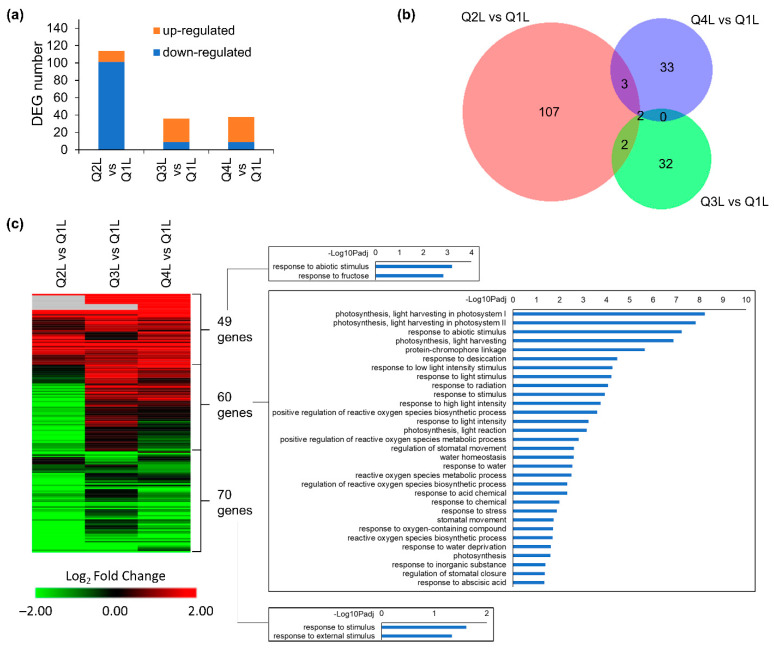
DEGs detected in at least one comparison of Q2, Q3 or Q4 vs. Q1 in leaves. (**a**) Number of DEGs in three comparisons. (**b**) Overlap between DEGs detected in each comparison. (**c**) Heatmap showing expression patterns of 179 DEGs detected in at least one comparison. GO terms of biological processes for three clusters of genes are shown. Hierarchical clustering of heatmap was done using one minus cosine similarity. Genes with no expression detected in RNA-seq are indicated in gray.

In leaf tissues (L), 114 DEGs were identified in the Q2L vs. Q1L comparison, followed by 36 DEGs in Q3L vs. Q1L, and 38 DEGs in Q4L vs. Q1L (Figure 5a). These results suggest that the leaf tissues do not have as much of a response to being in the KFTs when compared to the root tissues. The deceasing number of DEGs over time (Figure 5a), suggests that leaves might be adapting quickly to the KFT environment. From the number of DEGs seen in each comparison, a large proportion (>90%) were unique to each time-point, with very little overlap (Figure 5b). However, looking at the trends of the gene expression heatmap of all DEGs across all time points, several distinct clusters are observed (Figure 5c, Table S3). In the first cluster, genes were generally upregulated across all the time points and GO enrichment analysis indicated that these set of 49 genes were associated with responses to fructose. The second cluster of 60 genes showed varying regulation of gene expression across the time points with GO enrichments indicating responses to light and photosynthesis. The third cluster showed 70 genes that were generally downregulated across all the time points. GO enrichment showed genes that were downregulated in the third cluster were associated with responses to stimuli. Among these, light associated responses were striking and could be explained by the altered light conditions when plants were inserted into KFTs. The thickness and slight opaqueness of the material used to construct the KFT might cause a reduction of light reaching the plants. Overall, the hypoxic responses in roots and light-related signaling in leaves were the most observable influence on plants when grown in the KFT for a short period of time.

### 3.4. Short-Term Responses of Plants to Gravity Alteration in the KFT

To test whether the KFT setup can capture the plant responses to gravity changes with the appropriate controls, the transcriptome of plants grown in the KFTs, placed on the clinostat (C) from the T+0 to the T+8 min timepoint, were compared to a series of different controls. The controls used were: Q1 (T-10 min), Q3 (T+8 min) that has the exact duration of time within the KFTs as (C). Due to the nature of spaceflight experiments, especially in the current early stages of operational developments, such well-timed coordination may not always be feasible. Therefore, the Clinostat (C) sample was also compared to a pooled control of Q1 and Q3, and a combined control of Q1, Q2 and Q3.

**Figure 6:**
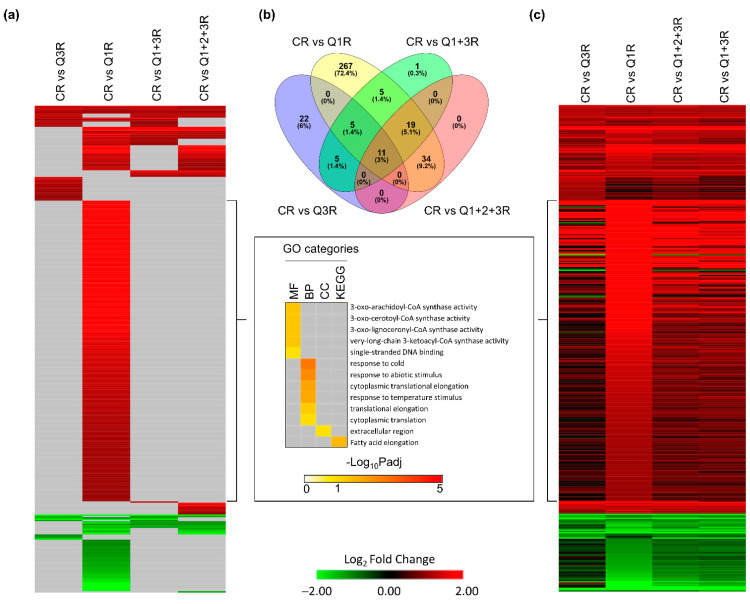
DEGs of clinostat treatment (C) compared with four different controls in roots. (**a**) Heatmap showing significant expression patterns of 369 DEGs detected in at least one comparison. Genes with −1 < Log2FC < 1, or Padj > 0.05, or no expression data detected in RNA-seq, are filled using gray. GO terms are shown for 238 significantly induced genes that are only detected using Q1. (**b**) Overlap between DEGs detected in each comparison. (**c**) Heatmap showing full expression patterns of 369 DEGs detected in at least one comparison. The order of genes is the same as (**a**). Genes with no expression detected in RNA-seq are indicated in gray.

Clinostat roots (CR) compared to the various root controls (Q3R, Q1R, combined Q1R + Q3R, and pooled Q1R + Q2R + Q3R) revealed that although gene expression trends were similar across all comparisons, using the appropriate controls can better segregate between gravity-related responses from other confounding responses (Figure 6). In root tissues, a total of 369 genes showed significant differential expression in at least one comparison (Figure 6a). In this experimental design, Q1 was designated as a sample point before “launch”. The root tissues in CR were in the KFT 18 min longer than Q1R and showed a substantial number of unique DEGs not observed in the other controls, which had GO enrichment mainly involved in temperature stimulus and abiotic stress responses (Figure 6a). The overlaps of the significant DEGs between each comparison showed 11 (3%) genes shared by all comparisons, 79 (21.4%) genes were shared by at least two comparisons, and 267 (72.4%) genes were unique to Q1R (Figure 6b). The gene expression trends showed that the majority of the 369 genes shared similar expression profiles regardless of the control used (Figure 6c, Table S4). Comparing the gene expression trends between CR versus Q1R and the other comparisons, there is an observable distinction of the foldchange properties of the 228 genes unique to Q1R. In comparing CR to Q3R and when controls were pooled (Q1R + Q3R and Q1R + Q2R + Q3R), these 228 genes no longer met the DEG classification thresholds (Figure 6c). These results demonstrated that the KFT setup successfully detected plant responses to gravity changes, though using the appropriate controls can help distinguish between the clinostat response and the likely accumulation of hypoxic stress and/or other environmental stimuli seen when using Q1R as a control (Figure 4).

**Figure 7:**
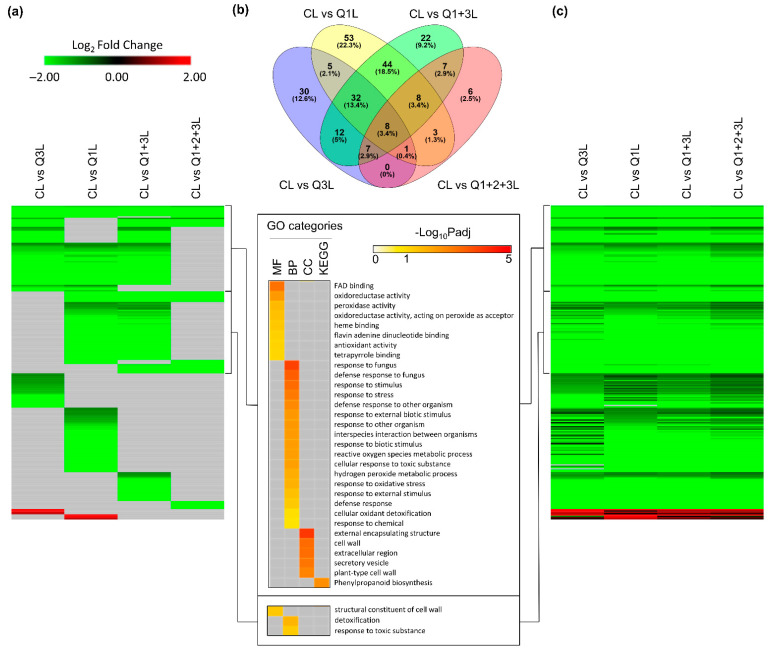
DEGs of clinostat treatment (C) compared with four different controls in leaves. (**a**) Heatmap showing significant expression patterns of 238 DEGs detected in at least one comparison. Genes with −1 < Log2FC < 1, or Padj > 0.05, or no expression data detected in RNA-seq, are filled using gray. GO terms are shown for two clusters of genes. (**b**) Overlap between DEGs detected in each comparison. (**c**) Heatmap showing full expression patterns of 238 DEGs detected in at least one comparison. The order of genes is the same as (**a**). Genes with no expression detected in RNA-seq are indicated in gray.

In leaves, the variations between the comparisons were not as pronounced as those seen in the roots but a similar trend was observed. A total of 238 DEGs were detected in at least one comparison (Figure 7a). Of these DEGs, a substantial number were shared across the various comparisons. The Venn diagram (Figure 7b) showed that 8 genes (3.4%) were shared across all comparisons, 127 (53.4%) genes were shared by at least two comparisons, and CL vs. Q1L had the highest number of unique genes at 22.3% (53 genes). GO enrichment of the first 65 genes in column one, Figure 7a, showed processes such as defense-related pathways and the next set of 65 genes showed an enrichment of pathways associated with cell wall and detoxification. These processes are typically observed in gravity-related responses of Arabidopsis in previous spaceflight-related transcriptomic analyses [20,21,22,23]. In leaves, the selection of an appropriate control does not seem to have as substantial an impact as roots on capturing the clinorotation response (Figure 7c, Table S5).

Taken together, when obtaining a well-coordinated control is not feasible, pooling controls from multiple time points can still successfully assay short-term spaceflight responses in plants. Pooled controls similarly reveal gene expression trends and eliminate other confounding responses as do the well-coordinated control, especially in roots which are known to be more sensitive to gas exchange alteration [24,25].

It is expected that the suborbital flight would encounter hyper-g profiles and vibrations that could impact the plant’s transcriptional response. Previously conducted suborbital flight experiments, using 7–12 day old plants grown on the same gel matrix described in this manuscript, have shown that plants adhere well to the gel matrix during the entire flight profile without sustaining any damages [26]. Thus, although clinorotation of the KFT has its limitations in simulating a suborbital flight, it does allow a glimpse of how researchers would still be able to capture gravity-associated responses using this method.

## 4. Conclusions

The increased accessibility of space is crucial for the advancement of science and technology that will enable humanity to meet the goals of living on the moon and Mars. Suborbital flights now allow civilian passengers to travel to space and back, presenting a unique opportunity for scientists to perform human-tended research. The intent of this investigation is to develop a method that will enable scientists to perform hands-on plant molecular biology experiments efficiently in the suborbital environment, thereby broadening the platforms from which investigators can conduct space-related experiments. KFTs have been used to fix samples in orbital space in a wider variety of biological systems for a decade or so, but have never been used as a vessel to house samples to space. The data presented herein suggest that KFTs can be readily adapted for carrying biological samples to space in a safely contained fashion. In addition, these data suggest that the changes in plant biology that do occur upon sealing within the KFTs are manageable within the flight timelines and experiment profile, provided that comprehensive ground controls are enacted. Plants grown in the thoroughly enclosed KFTs can maintain consistent plant health for several hours, thus, providing ample time to prepare and conduct a human-tended experiment onboard the suborbital flight. Moreover, plants within KFTs exhibited robust changes in gene expression when clinorotated during the simulated microgravity portion of a suborbital flight, suggesting the potential for capturing transcriptomic responses to the actual high g and microgravity portions of a suborbital flight. This method of prepping biology to be housed in KFTs and then using KFTs for suborbital flight experiments should provide a tractable initialization of hands-on research in suborbital space.

## Figures and Tables

**Table 1:** Number of DEGs in the pairwise comparison of four KFTs in roots.

Comparison (Treatment vs. Control)	Total DEG	Upregulated	Downregulated
Q1B vs. Q1A	1	1	0
Q1C vs. Q1A	4	2	2
Q1D vs. Q1A	16	4	12
Q1C vs. Q1B	7	3	4
Q1D vs. Q1B	94	22	72
Q1D vs. Q1C	17	6	11
Average	23	6	17

## Data Availability

Illumina Sequencing data have been deposited to the Gene Expression Omnibus repository under the National Center for Biotechnology Information database. The accession number GSE214377 has been assigned to the data used in this manuscript.

## Supplementary Materials

The following supporting information can be downloaded at Zenodo: https://zenodo.org/record/7129570#.YzcBT3bMKF4 (accessed on 11 November 2022)
